# Embracing JMA Journal: A Small Wish from a Seasoned Reader and Author

**DOI:** 10.31662/jmaj.2024-0151

**Published:** 2024-08-09

**Authors:** Shigeki Matsubara

**Affiliations:** 1 Department of Obstetrics and Gynecology, Jichi Medical University, Tochigi, Japan; 2 Department of Obstetrics and Gynecology, Koga Red Cross Hospital, Koga, Japan; 3 Medical Examination Center, Ibaraki Western Medical Center, Chikusei, Japan

**Keywords:** case report, editor, human thoughts, impact factor, article processing charge

## Dear Editors:

The editorial by Professor Tsuguya Fukui, Editor-in-Chief of the Journal, announced that the JMA Journal gained an Impact Factor (IF) of 1.5 and will provide a worldwide medical platform ^(1)^. I sincerely thank everyone whose efforts made this possible.

I have some aspirations. First, I hope that the JMA Journal continues to charge no article processing charges (APC). Transitioning to open access with APC increases the journal IF ^(2)^. After retiring from university, I recognized that affording APC is challenging without funding. Open access with APC deprives those with limited financial backgrounds of the chance to submit manuscripts ^(3)^.

Second, I request that the JMA Journal continue to accept case reports. The value of the paper does not depend on article categories ^(4)^. Case reports often provide important information. At present, only a few medical journals with international leadership accept case reports, considering that the publication of case reports could hamper the increasing IF. Case reports do not need to be long; some journals publish case reports under Letters. We―readers and authors―agree that the level of acceptance of case reports could be high. I believe that short, attractive, and scientifically meaningful case reports can be a fortune for the JMA Journal.

Third, progress should not be based solely on objective data. Personal concepts or thoughts, especially those based on lifelong experiences, provoke readers’ thoughts and contribute to future academic progress. JAMA has a “Poem” section, indicating that human thoughts, regardless of their less evidence-based aspects, are respected. I wish that the JMA Journal continues to publish these short and brilliant pieces.

At present, many journals target subspecialties. This tendency may reflect the current trend: medicine has become more specialized. To deepen the specific area, narrowing the theme may merit readers of the area. However, medicine treats people with general rather than specific conditions and problems. Irrespective of one’s specialties, we must know about general medicine. The JMA Journal has been providing up-to-date general knowledge.

On July 4^th^, 2024, new banknotes were delivered. Shibasaburo Kitasato, an esteemed Japanese physician-researcher, appeared in a 1,000-yen bill, making us self-confident. Japanese medicine runs the world front. Professor Fukui touched on JAMA and BMJ. I hope that JMA Journal will be added to the current “Big Four,” constituting a “Big Five.” This is not a courtesy. I, a seasoned Japanese doctor, believe so.

## Article Information

### Conflicts of Interest

None

### Author Contributions

S. Matsubara: Identification of the significance and manuscript writing.

### Approval by Institutional Review Board (IRB)

Not applicable

### Patient Anonymity

Not applicable

### Informed Consent

Not applicable

### Data Availability

Data sharing is not applicable to this article, as no new data were created or analyzed in this study.
